# The AMC Linear Disability Score project in a population requiring residential care: psychometric properties

**DOI:** 10.1186/1477-7525-2-42

**Published:** 2004-08-03

**Authors:** Rebecca Holman, Robert Lindeboom, Marinus Vermeulen, Rob J de Haan

**Affiliations:** 1 Department of Clinical Epidemiology and Biostatistics, Academic Medical Center, Amsterdam, The Netherlands; 2 Department of Neurology, Academic Medical Center, Amsterdam, The Netherlands

## Abstract

**Background:**

Currently there is a lot of interest in the flexible framework offered by item banks for measuring patient relevant outcomes, including functional status. However, there are few item banks, which have been developed to quantify functional status, as expressed by the ability to perform activities of daily life.

**Method:**

This paper examines the psychometric properties of the AMC Linear Disability Score (ALDS) project item bank using an item response theory model and full information factor analysis. Data were collected from 555 respondents on a total of 160 items.

**Results:**

Following the analysis, 79 items remained in the item bank. The remaining 81 items were excluded because of: difficulties in presentation (1 item); low levels of variation in response pattern (28 items); significant differences in measurement characteristics for males and females or for respondents under or over 85 years old (26 items); or lack of model fit to the data at item level (26 items).

**Conclusions:**

It is conceivable that the item bank will have different measurement characteristics for other patient or demographic populations. However, these results indicate that the ALDS item bank has sound psychometric properties for respondents in residential care settings and could form a stable base for measuring functional status in a range of situations, including the implementation of computerised adaptive testing of functional status.

## Background

It is now widely accepted that examining quality of life is an important aspect in the treatment and evaluation of many conditions. Functional status is seen as an important determinant of quality of life. A wide variety of instruments have been developed to quantify functional status [1]. These instruments tend to have a fixed length and all items are administered to the whole group of patients under scrutiny. However, currently interest is moving towards the more flexible framework offered by item banks. An item bank is a collection of items, for which the measurement properties of each item are known [2,3]. When using an item bank, it is not essential for all respondents to be examined using all items. This enables the burden of testing to be considerably reduced for both patients and researchers. It is even possible to select the 'best' items for individual patients using computerised adaptive testing algorithms [4]. Furthermore, results from studies using different selections of items from an item bank can be directly compared. Item banks, measuring concepts such as quality of life [2,5], the impact of headaches [6] or functional status [7,8], have been developed.

The AMC Linear Disability Score (ALDS) project item bank was developed to quantify functional status [7,9]. The ALDS item bank covers a large number of activities, which are suitable for assessing respondents with a very wide range of functional status and many types of chronic condition. The item bank is particularly suitable for use in the Netherlands. The ALDS items were obtained from a systematic review of generic and disease specific functional health instruments [1]. Five psychometric aspects of the ALDS item bank need to be considered before it can be implemented. These are: (a) there needs to be enough variation in the response categories used for each item [9]; (b) estimates of the item response theory model parameters should not depend on patient characteristics such as age or gender [10,11]; (c) estimates of the item response theory model parameters, which are stable across different subsets of items from the instrument and based on a sufficiently large sample [12] of respondents, should be available [9]; (d) an examination of the extent to which the ALDS items represent a single construct; and (e) testing whether a simpler item response theory model is suitable for the set of items.

This paper examines these five aspects of the ALDS item bank using the responses given by residents of supported housing schemes, residential care and nursing homes in and around Amsterdam, the Netherlands. This, mainly elderly, population has been chosen because they generally experience some level of functional restriction and consume a large amount of health care services.

## Methods

### Data collection

This paper considers 160 items, which were considered to be applicable in a residential care setting. Each item has two response categories: 'I could carry out the activity' and 'I could not carry out the activity'. If a respondent had never had the opportunity to experience an activity 'not applicable' was recorded. In the analysis, responses in the category 'not applicable' were treated as if the individual items had not been presented to the individual respondents [13]. It was felt that presenting all 160 items to each respondent would place an unnecessary and unacceptable burden on those responding to the items. Therefore, the data described in this paper were collected using an incomplete, anchored calibration design [7,9,14,15] with four sets of 80 items. Item sets *A *and *B *have half their items in common, as do item sets *B *and C, item sets *C *and *D *and item sets *A *and *D*. The items in common between two sets of items are known as 'anchors' and allow all items and patients to be calibrated on the same scale. The patterns of missing data in this type of design are, in statistical terms, ignorable [16]. The item sets were administered randomly to 150 respondents (item set *A*), 143 respondents (item set *B*), 138 respondents (item set *C*) and 124 respondents (item set *D*).

### Respondents

A total of 555 residents of supported housing, residential care and nursing homes were interviewed. The median age was 84 years (range 37 to 101 years), while 444 (80%) were female. Since the respondents were interviewed 'at home', accurate data on medical conditions were not available. All respondents gave informed consent. The study was approved by the medical ethics committee in our hospital.

### The item response theory models

In this paper the data were analysed using the two-parameter logistic item response theory model [7,9,17,18]. In this model, the probability, *P*_*ik*_, that patient *k *responds to item *i *in the category 'can' is modelled using



where *θ*_*k *_denotes the ability of patient *k *to perform activities of daily life. The discrimination parameter (*α*_*i*_) and difficulty parameter (*β*_*i*_) describe the measurement characteristics of item *i*. The larger the value of *β*_*i*_, the more difficult item *i *is. In addition, the larger the value of *α*_*i*_, the better an item is a distinguishing between abilities above and below *β*_*i*_. If the values of *α*_*i *_are constrained to be equal for all items, the model in equation 1 becomes the one-parameter logistic item response theory model [19]. The model in equation 1 can be extended to test whether the values of *β*_*i *_for, say males and females, are significantly different. If the values of *β*_*i *_for different groups of respondents are significantly different, then there is evidence of differential item functioning. Full-information factor analysis also uses an extension of the model in equation 1. These approaches are described in mathematical terms in the Appendix. In this paper, estimates of *α*_*i *_and *β*_*i *_were obtained using a marginal maximum likelihood based procedure [20]. This method assumes that the ability parameters (*θ*_*k*_) follow a Normal distribution and can account for incomplete designs, as described in the Appendix. Expected a posteriori methods were used to estimate *θ*_*k *_[21].

### Statistical analysis

To achieve the objectives of this study, there were five steps in the statistical analysis. In step (a), the amount of variation in the response categories used for each item [9] was considered and items demonstrating too little variation were removed. Items were excluded from further analysis if fewer than 10% or more than 90% of the patients responded in the category 'cannot'. In step (b), the items were examined to investigate whether the value of the item difficulty parameter (*β*_*i*_) was similar for male and female patients and for patients younger than 85 years and those aged 85 or older. The model is described in depth in the Appendix. Items were excluded from further analysis if the value of the item difficulty parameter was significantly different (1% level) between gender or aged based groups. In this step, the fit of the model to the data from each item was not assessed. In step (c), estimates of the item parameters (*α*_*i *_and *β*_*i*_) were obtained. The fit of the model to the data from each item was assessed using *G*^2 ^statistics [22]. Items, for which the fit statistic had a *p*-value of less than 0.01, were excluded from the item bank. In addition, the stability of the estimates of the item parameters over different sets of items was examined using the model from step (b). Items were excluded from further analysis if the value of the item difficulty parameter was significantly different (1% level) between item sets *A *and *B, B *and *C, C *and *D *or *A *and *D*. Furthermore, a Kolmogornov-Smirnov test was carried out to examine whether the ability parameters (*θ*_*k*_) were Normally distributed. In step (d), the dimensionality of the item bank was examined using item response theory based full information factor analysis [18,22,23]. The number of latent roots greater than 1 is regarded as an indicator of the number of factors in the data set. This method is described in more depth in the Appendix. Four exploratory factor analyses were carried out, one on each of the anchors between item sets *A *and *B *(293 respondents), *B *and *C *(281 respondents), *C *and *D *(262 respondents) or *A *and *D *(274 respondents). A fifth, confirmatory, factor analysis was carried out on the whole data set (555 respondents). In addition, Cronbach's coefficient alpha was calculated for each anchor and the whole data set [24,25]. In step (e) the one-parameter logistic item response theory model was fitted to the remaining items. The differences between the -2log likelihoods of this model and the two-parameter model fitted in step (c) was tested using a *χ*^2 ^test. The analysis in steps (a), (b), (c) and (e) was carried out in Bilog, version 3.0 [22]. The analysis in step (d) was carried out using TESTFACT, version 4.0 [22].

## Results

Of the 160 items included in the item bank, one was removed because it was worded differently in two different item sets. Of the 159 remaining items, 77 were removed from the item bank. This process is described in Table 1. In step (a), 28 items were excluded from further analysis because fewer than 10% or more than 90% of responses were in the category 'cannot'. In step (b), 26 items were removed because they had significantly different estimates of the item difficulty parameter (*β*_*i*_) for for males and females and/or for younger and older respondents. Of these 26 items, 19 had different measurement characteristics for females and for males, 5 items had different measurement characteristics for those aged under 85 and for those aged 85 or over, and 2 items had different measurement characteristics for both males and females and for older and younger respondents. In step (c), 23 items had an item fit statistic *p*-value of less than 0.01. In addition, 3 items were excluded from further analysis because the value of the item difficulty parameter (*β*_*i*_) was significantly different between two item sets of items. Hence, 79 psychometrically sound items remained in the item bank. A short description of the content of the 79 items in the final version of the calibrated item bank, together with estimates of the dispersion (*α*) and difficulty (*β*) parameters and their standard errors, are given in Tables 2a and 2b. Following step (c) of the analysis, the anchors between the sets of items contained between 13 and 23 items. In addition, there was no evidence to suggest that estimates of *θ *do not follow a Normal distribution (Kolmogorov-Smirnov test, *p*-value = 0.637). In step (d), the full information factor analysis indicated that, for three of the four anchors between the item sets, there was only one latent root of the correlation matrix larger than 1. In the fourth item set, a second latent root was marginally above 1. The percentage of the variance explained by the first factor varied between 67% and 72%. The values of Cronbach's alpha coefficient for the four anchors were between 0.86 and 0.93. The confirmatory factor analysis carried out on the whole data set indicated that 70% of the variance was explained by the first factor. Cronbach's alpha coefficient for the whole data set equalled 0.98. In step (e), the one-parameter logistic item response theory model was fitted to the 79 items remaining after step (c). This model fitted the data significantly less well than the two-parameter model (*p*-value < 0.0001). For 3 items, the item fit statistic had *p*-value < 0.01. After removal of these items, the two-parameter model was still significantly better than the one-parameter model (*p*-value < 0.0001).

**Table 1:** The number of items proceeding to each step of the analysis The number of and examples of items removed at each stage of the psychometric analysis.

Stage of analysis	Number of items removed	Reason for removal	Examples
	1	Concerns about the way the item was presented	
(a)	28	< 10% or > 90% of responses in 'cannot'	Reaching for a cup and taking a sip of water Combing hair at a sink Cycling on a heavily laden bicycle
(b)	26	Significant difference between M and F and/or under and over 85 years	Washing up (easier for older respondents) Crossing the street (easier for younger respondents Preparing a warm meal (easier for female respondents)
(c)	26	Item fit *p*-value < 0.01 or estimates of *β*_*i *_not stable	Taking oral medication Cycling Getting money out of the bank using an ATM
In item bank	79		See Table 2
Total	160		

**Table 2:** The 79 items remaining in the calibrated item bank. The items remaining in the calibrated item bank. The number of respondents, to whom the item was offered (Offered to), the number responding in the category 'not applicable' (NA), the number responding in the category 'can' (can) and the number responding in the category 'cannot' (cannot) are given. The discrimination (*β*) and difficulty (*β*) parameters are given along with their standard errors in parentheses.

Description of item content	Offered to	Item response category			Location parameter (*β*)		Discrimination parameter (*α*)	
		NA	can	cannot				
Walking up stairs with a bag	262	0	19	243	-3.607	(2.404)	1.122	(0.892)
Mopping a flight of stairs	262	5	16	241	-2.830	(1.708)	0.447	(0.411)
Cleaning the top of a high cupboard	281	2	27	252	-2.816	(1.946)	0.480	(0.393)
Cleaning a bathroom	293	1	37	255	-2.621	(2.061)	0.323	(0.338)
Vacuuming	274	0	33	241	-2.408	(1.844)	0.287	(0.280)
Going for a walk in the woods	281	0	31	250	-2.343	(1.636)	0.362	(0.340)
Fetching groceries for 3–4 days	293	0	36	257	-2.262	(1.623)	0.353	(0.343)
Mopping the floor	281	2	41	238	-2.225	(1.902)	0.339	(0.374)
Caring for plants on a balcony	262	1	32	229	-2.108	(1.616)	0.314	(0.325)
Travelling by bus or tram	281	0	44	237	-2.093	(1.835)	0.308	(0.370)
Walking up two flights of stairs	274	0	39	235	-1.921	(1.532)	0.277	(0.298)
Cleaning a fridge	293	2	64	227	-1.406	(1.464)	0.171	(0.236)
Going to a restaurant	293	3	60	230	-1.335	(1.238)	0.159	(0.188)
Carrying a tray	281	1	75	205	-1.304	(1.774)	0.222	(0.326)
Going for a long walk (15+ minutes)	281	0	72	209	-1.290	(1.629)	0.178	(0.277)
Going to the dentist	293	18	75	200	-1.283	(1.881)	0.205	(0.299)
Sweeping the floor	262	1	70	191	-1.239	(1.902)	0.196	(0.313)
Cutting toe nails	262	0	45	217	-1.175	(0.786)	0.136	(0.144)
Walking up a hill or bridge	281	3	73	205	-1.133	(1.425)	0.137	(0.198)
Walking up one flight of stairs	274	2	67	205	-1.127	(1.382)	0.155	(0.222)
Going to a concert	262	0	57	205	-0.996	(0.860)	0.113	(0.128)
Going to the pharmacist	262	2	75	185	-0.976	(1.572)	0.131	(0.201)
Hanging a load of washing out	293	10	84	199	-0.960	(1.514)	0.144	(0.240)
Going to the post office or bank	274	0	88	186	-0.948	(1.881)	0.151	(0.248)
Going to a party	281	1	69	211	-0.924	(0.878)	0.109	(0.129)
Filling an official form in	281	1	68	212	-0.896	(0.790)	0.106	(0.119)
Using a washing machine	281	6	95	180	-0.851	(1.743)	0.153	(0.244)
Visiting an outpatients' clinic	293	0	95	198	-0.815	(1.317)	0.112	(0.161)
Taking bottles to the bottle bank	281	5	108	168	-0.675	(1.840)	0.153	(0.312)
Short walk (less than 15 minutes)	274	0	95	179	-0.645	(1.358)	0.108	(0.166)
Putting a rubbish bag outside	293	5	108	180	-0.573	(1.338)	0.116	(0.198)
Reaching into a high cupboard	274	0	95	179	-0.569	(1.097)	0.099	(0.172)
Using a dustpan and brush	262	2	103	157	-0.537	(1.865)	0.135	(0.335)
Opening and closing a high window	281	0	140	141	-0.078	(1.290)	0.096	(0.166)
Fetching groceries for one day	262	0	128	134	-0.043	(1.373)	0.099	(0.184)
Using a public toilet	293	5	159	129	0.139	(1.835)	0.110	(0.241)
Putting flowers in a vase	293	2	162	129	0.169	(1.787)	0.107	(0.240)
Frying an egg	281	3	154	124	0.178	(1.982)	0.115	(0.261)
Warming up a tin of soup	293	1	164	128	0.203	(1.919)	0.113	(0.232)
Cleaning a toilet	262	0	149	113	0.308	(1.528)	0.105	(0.203)
Putting socks and lace up shoes on	281	1	167	113	0.314	(1.165)	0.093	(0.157)
Changing the bulb in a table light	281	1	177	103	0.533	(1.541)	0.116	(0.174)
Cleaning a bathroom sink	281	5	170	106	0.564	(2.089)	0.126	(0.302)
Cutting finger nails	262	0	168	94	0.605	(1.337)	0.114	(0.173)
Rubbing lotion into whole body	262	4	164	94	0.627	(1.469)	0.115	(0.184)
Reaching into a low cupboard	274	0	184	90	0.672	(1.131)	0.106	(0.152)
Picking something up off the floor	262	0	172	90	0.712	(1.466)	0.129	(0.198)
Making porridge	293	2	191	100	0.714	(1.704)	0.119	(0.216)
Getting in and out of a car	281	3	185	93	0.738	(1.656)	0.132	(0.209)
Shaking a tablecloth out	274	2	190	82	0.906	(1.438)	0.125	(0.204)
Making a bed	281	0	193	88	1.003	(2.028)	0.152	(0.292)
Preparing breakfast or lunch	262	1	186	75	1.117	(1.729)	0.169	(0.279)
Using the lift in a public building	262	2	199	61	1.208	(1.299)	0.158	(0.186)
Putting an alarm clock right	281	4	216	61	1.319	(1.431)	0.165	(0.199)
Pulling a blanket up	293	0	253	40	1.485	(0.898)	0.167	(0.149)
Visiting the neighbours	293	1	231	61	1.548	(1.685)	0.221	(0.252)
Travelling as a passenger in a car	274	3	230	41	1.592	(1.126)	0.222	(0.192)
Shaving face or applying make up	274	1	233	40	1.593	(1.075)	0.180	(0.164)
Watering a house plant	262	3	204	55	1.600	(1.681)	0.226	(0.259)
Opening and closing a window	262	0	201	61	1.735	(2.137)	0.246	(0.343)
Putting trousers on	293	2	224	67	1.821	(2.372)	0.295	(0.406)
Making coffee or tea	293	0	235	58	1.832	(1.936)	0.237	(0.290)
Peeling an apple	281	1	233	47	1.859	(1.631)	0.226	(0.219)
Making a bowl of cereal	281	1	225	55	1.860	(1.921)	0.222	(0.256)
Eating a meal at the table	293	0	255	38	2.081	(1.509)	0.253	(0.225)
Hanging clothes up in a cupboard	262	0	203	59	2.105	(2.595)	0.344	(0.481)
Opening and closing curtains	262	0	216	46	2.129	(1.958)	0.366	(0.357)
Moving between two dining chairs	281	0	237	44	2.214	(1.905)	0.389	(0.375)
Putting a scarf and gloves on	293	1	259	33	2.364	(1.617)	0.306	(0.246)
Making a cheese sandwich	281	1	243	37	2.416	(1.856)	0.392	(0.333)
Moving to sit on the edge of a bed	262	1	231	30	2.457	(1.658)	0.309	(0.244)
Putting a coat on	274	0	227	47	2.463	(2.323)	0.425	(0.395)
Putting a shirt or blouse on	262	0	228	34	2.495	(1.842)	0.360	(0.287)
Washing upper body at a sink	274	0	243	31	2.705	(1.875)	0.420	(0.327)
Answering the front door	274	1	233	40	2.792	(2.373)	0.481	(0.449)
Getting out of bed into a chair	262	0	232	30	3.019	(2.132)	0.581	(0.448)
Washing lower body at sink	293	1	241	51	3.037	(3.098)	0.722	(0.761)
Putting a T-shirt on	293	2	257	34	3.440	(2.664)	0.718	(0.630)
Locking a door	262	0	230	32	3.366	(2.512)	0.970	(0.749)

NA denotes that the category 'not applicable' was chosen

## Discussion

In this study, the psychometric properties of the item bank have been examined using a sample of 555 respondents and an incomplete calibration design. Each item was presented to between 262 and 293 respondents. These figures are above the minimum, of 200 respondents, regarded as necessary to implement the models used in this paper [12]. It could be argued that it would have been desirable for all respondents to be presented with all items, but this would have placed an unacceptable burden on the, often frail, population in this study. Incomplete calibration designs are regularly implemented in the development and maintenance of item banks used in educational testing [4,14] and have gained some recognition in health related applications [15]. Developments in psychometric theory mean that it is now possible to perform the same types of analysis on data resulting from incomplete designs, as is performed on data from complete calibration designs [22,23,25]. The number of items in the anchors following the analysis, indicate that the design was still amply linked [9].

One of the major assumptions underlying the use of the item response theory models described in this paper is that the items reflect a single latent trait (*θ*). This has been examined using item response theory based full-information factor analysis. Part of the full-information factor analysis was performed on sub-sets of the data, as exploratory analyses on incomplete designs may lead to instable results. However, the confirmatory factor analysis was performed on all data. The results, together with the high level of internal consistency, as measured by Cronbach's alpha, and the acceptable fit of the two-parameter logistic item response theory model to the data indicate that the items presented in this paper probably represent a unidimensional construct in a population of respondents requiring residential care.

Another important assumption when using item response theory models in conjunction with marginal maximum likelihood estimation procedures is that the values of the latent trait (*θ*) follow a pre-specified, usually Normal, distribution. In this study, there was no evidence that these values did not follow a Normal distribution. This is in contrast to many previously published studies into health and quality of life outcomes, where a strongly skewed distribution was found. The authors feel that there are two reasons for this contrast. Firstly, in this study, the respondents all had some level of restriction in their ability to perform activities of daily life. Secondly, the item bank includes items well above and well below the level of functional status enjoyed by the respondents. This means that the item bank did not have a ceiling or floor effect with respect this this population.

In this study, 81 (51%) of 160 items were removed from the item bank because they did not conform to the psychometric standards required of the item bank. This is a much higher level than would be expected in the calibration of an item bank for use in educational measurement. However, when the results are examined more carefully, 28 items were removed because they were too difficult or too easy for the population in this study. In addition, 26 items were removed because they had different item parameters for different groups of respondents. These problems would have been identified much earlier in an educational item bank. Hence, only 26 (25%) of 106 items were removed due to item misfit. The number of items retained in the item bank may have been higher if a more flexible model, based on, for example, non-parametric smoothing techniques had been used [26]. However, this type of model is less suitable as a base for implementing modern testing algorithms, such as computerised adaptive testing. In addition, it is possible that more items could be made available if the items demonstrating differential item functioning were included in the item bank with different item location parameters (*β*_*i*_) for males and females or for younger and older respondents. This may seem complicated, but is straightforward in the framework of a computerised item bank.

This paper has concentrated on the two-parameter logistic item response theory model. However, the one-parameter logistic item response theory model was also fitted to the 79 items remaining in the item bank. This model fitted the data significantly less well than the two-parameter model, even after 3 items demonstrating misfit at the item level were removed. This confirms the choice of the two-parameter model. This model was chosen because it allows the probability of responding in the category 'can' to be modelled more flexibly than when the one-parameter logistic model is used. This enables a more realistic model for the data to be built than when the more restrictive approach associated with the one-parameter model is chosen [18].

This paper has examined the calibration of the ALDS item bank in a population requiring residential care. It has been shown that the item bank has sound psychometric properties and could form a stable base for a wide range of applications. However, it is possible that the items will have different measurement characteristics for patients requiring treatment for specific chronic conditions or in other countries. Hence, it is important that the ALDS item bank is tested carefully before it is used to assess the functional status in other groups of respondents or in other countries.

## Conclusions

Now that the measurement properties of the ALDS item bank have been examined carefully, the item bank can be used as a foundation for quantifying functional status. If modern algorithms, such as computerised adaptive testing, are implemented, it will be possible to obtain accurate measurements, whilst keeping the burden of testing on respondents and interviewers to a minimum. Items can be selected for use in further research, for allocation individuals to appropriate care settings and for calculating institutional funding based on the actual care load. It is hoped that the ALDS item bank will play an important part in the implementation of computerised adaptive testing of functional status.

## Appendix

### Differential item functioning

The model in equation 1 can be extended to test whether different groups of respondents to have different values of *β*_*i*_. This is known as differential item functioning. For instance, if interest is in possible differences in *β*_*i *_between males and females, then the probability, *P*_*ik*_, that patient *k *responds to item *i *in the category 'can' is written as



where *β*_*iM *_is the item difficulty for male respondents, *β*_*iF*-*M *_is the difference between the item difficulty for males and for females and *I*_*k *_is an indicator variable taking the value 0 if respondent *k *is male and the value 1 if respondent *k *is female. The hypothesis H_0 _: *β*_*iF*-*M *_= 0 can be tested to examine whether item *i *has the same measurement characteristics for males and for females.

### Item parameter estimation in incomplete designs

In this study, the item parameters (*α*_*i *_and *β*_*i*_) were estimated using marginal maximum likelihood methods. The likelihood, *L*, over *n *items and *K *(*K *= 555) respondents can be written as



where *I*_*ik *_is an indicator variable taking the value 1 if respondent *k *was offered item *i *and the value 0 otherwise and where *J*_*ik *_is an indicator variable taking the value 1 if respondent *k *responded to item *i *in the category 'can' and the value 0 otherwise. Furthermore, the probability, *P*_*ik*_, that respondent *k *responded to item *i *in the category 'can' is as in equation 1, or, where appropriate, as in equation 2 or 4. In the estimation process, the values of *θ*_*k *_or *θ*_*km *_were assumed to follow a Normal distribution with mean equal to 0 and unknown variance, *σ*^2^, and were integrated out of the likelihood to obtain the marginal likelihood. The marginal likelihood was maximised using an EM algorithm [20].

### Full information factor analysis

Full information factor analysis is a technique based on multidimensional item response theory models where the ability is represented by *M *variables, denoted *θ*_*km *_where *m = *1, 2,..., *M *[22,23]. The model, in equation 1, for the probability, *P*_*ik*_, that person *k *responds to item *i *in the category 'can' can be extended to



where *θ*_*km *_denotes the value of the latent variable *θ*_*m *_associated with person *k *and *α*_*im *_denotes the discrimination parameter for item *i *with respect to the latent variable *θ*_*m*_. Furthermore, *δ*_*i *_is a difficulty type parameter. The loading, *a*_*im *_of item *i *on factor *m *can be calculated using



The value of the standard difficulty parameter, (*β*_*i*_), can be calculated using



Generally, the parameters *α*_*im *_and *δ*_*i *_are estimated using marginal maximum likelihood methods.

## Abbreviations

ALDS = AMC Linear Disability Score

## Competing interests

None declared.

## Funding

RH and RL were supported by a grant from the Anton Meelmeijer fonds, a charity supporting innovative research in the Academic Medical Center, Amsterdam, The Netherlands.

## Authors contributions

RL conceived the study and supervised the data collection. RH prepared the first draft and carried out the analyses. RL, MV and RJdH critically reviewed the manuscript. RH prepared the final version.
